# Oxidative Status as an Attribute for Selective Antitumor Activity of Platinum-Containing Nanoparticles against Hepatocellular Carcinoma

**DOI:** 10.3390/ijms232314773

**Published:** 2022-11-25

**Authors:** Kamil Wawrowicz, Agnieszka Majkowska-Pilip, Marzena Szwed, Kinga Żelechowska-Matysiak, Ewelina Chajduk, Aleksander Bilewicz

**Affiliations:** 1 Centre of Radiochemistry and Nuclear Chemistry, Institute of Nuclear Chemistry and Technology, Dorodna 16 St., 03-195 Warsaw, Poland; 2 Department of Nuclear Medicine, Central Clinical Hospital of the Ministry of the Interior and Administration, Wołoska 137 St., 02-507 Warsaw, Poland; 3 Department of Medical Biophysics, Faculty of Biology and Environmental Protection, University of Lodz, Pomorska 141/143 St., 90-236 Lodz, Poland; 4 Laboratory of Nuclear Analytical Techniques, Institute of Nuclear Chemistry and Technology, Dorodna 16 St., 03-195 Warsaw, Poland

**Keywords:** platinum nanoparticles, oxidative stress, hepatocellular carcinoma, breast cancer, anticancer therapy, spheroids

## Abstract

Overcoming the limitations for efficient and selective drug delivery is one of the most challenging obstacles for newly designed anticancer agents. In this study, we present two types of platinum-based nanoparticles (NP), ultrasmall 2 nm PtNPs and core-shell 30 nm Au@Pt, which can be highly cytotoxic in an oxidative environment and remain biologically inactive in cells with lower oxidative status. Our research highlighted the differences in platinum nanoparticle-induced chemotoxicity and is the first study examining its mechanism as a substantial aspect of Au@Pt/PtNPs biological activity. Selectively induced oxidative stress was found to be a primary trigger of NPs’ toxicity. Significant differences between Au@Pt and PtNPs were observed especially during 24 h treatment, due to successful intranuclear PtNPs location (~13% of internalized fraction). Reactive oxygen species (ROS)-level induced from both NPs types were similar, while reduction of reduced glutathione (GSH) intracellular content was stronger after treatment with PtNPs. Any biological activity was found in HER2+ breast cancer cells, which have only slightly increased oxidative status. Platinum-containing nanoparticles are an interesting tool for the improvement of selectivity in anticancer therapies against hepatocellular carcinoma (HCC). Due to intranuclear uptake, 2 nm PtNPs seems to be more promising for further research for HCC therapy.

## 1. Introduction

Platinum application as an anticancer agent is widely known, especially due to cis-platinum and its derivatives. The usefulness of complexed platinum drugs is severely limited by serious systemic toxicity that is induced in a dose-dependent manner [1]. The development of new nanoplatforms, which could modify the biological disposition of Pt compounds, reduce its toxicity and perhaps attenuate the undesirable side effects, is one of the most challenging tasks in experimental oncology to date [2].

**Figure 1:**
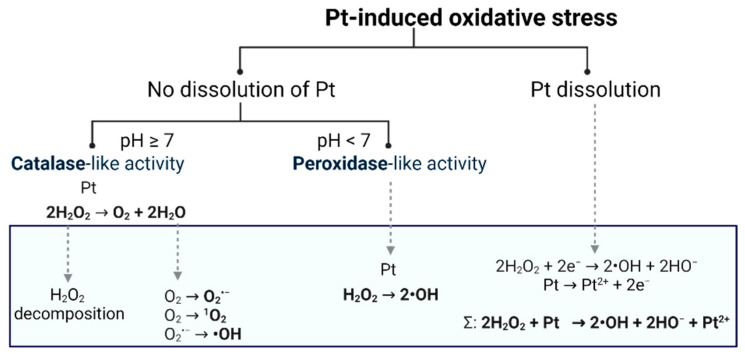
Postulated mechanisms of in vitro platinum interactions with H_2_O_2_. If lack of dissolution is considered, depending on pH, platinum may show catalase-like (pH ≥ 7) or peroxidase-like (pH < 7) activities. If dissolution of platinum is achieved, beyond pro-oxidative activity, cytotoxicity improvement is accomplished via formation of nuclear envelope penetrating cation—Pt^2+^. Each of the considered routes leads to the induction of oxidative stress via ROS formation.

As reported in [3], platinum becomes substantially more cytotoxic when it is downsized to nanoparticles (NPs). Thanks to their unique properties (e.g., large surface area in relation to mass and high reactivity), Pt nanoparticles have been rapidly recognized as nanomedicines which could be used in chemotherapy treatment of various types of cancer. Platinum may gain a catalytic function and consequently behave as an initiator of oxidative stress via reactive oxygen species (ROS) production, inducing significant damage leading to cell death. However, catalytic activity of platinum in vitro is highly dependent on oxidative status in cytosol, as only high-redox status resulting from increased H_2_O_2_ production enables it to trigger its reactivity. Thus, the enhancement of selectivity with the use of cytosolic properties of cancerous cells and the development of more effective anticancer agents can be achieved. Nevertheless, Pt biological activity in a high-oxidative-stress environment is yet to have a suitable mechanism of action uncovered. As shown in Figure 1, the solubility of Pt and environmental pH are two of the main factors that determine Pt interaction with hydrogen peroxide [4]. This implicates further alterations, especially concerning platinum dissolution, as a major determinant of therapeutic expectations.

**Figure 2:**
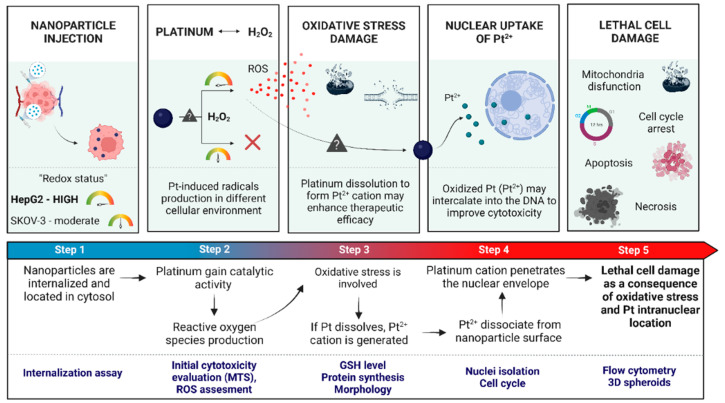
Investigated concept of platinum-containing nanoparticles as selective anticancer agent for treatment of “highly oxidative” cancers. After internalization, platinum may undergo reactions with H_2_O_2_ via different mechanisms (Figure 1). This may lead to reactive oxygen species (ROS) production and partial platinum oxidation to Pt^2+^. Subsequently, significant therapeutic effects can be observed by reduction of cell viability, apoptosis/necrosis and morphological changes in treated cells.

Since it is still unclear which oxidative pathway is mainly involved in the mechanism of action for Pt-based nanoparticle-sized molecules, this study aimed to investigate Pt-containing nanoparticles’ cytotoxicity, with a particular attention to cellular redox imbalance in vitro. Therefore, we focused on HCC and HER2+ breast/ovarian cancer cells. HepG2 cells are commonly known to be a cancer cell line with a high degree of oxidative stress, while SKOV-3 cells are characterized as having intermediately increased concentrations of cytosolic H_2_O_2_. Different redox status in these cell lines is desirable for a rational assessment of the environmental impact on the catalytic activity of platinum. We present the effects of Au@Pt/PtNP on cancer cell cytotoxicity and show that it depends on the redox homeostasis of cancer cell lines (Figure 2). Considering that surface-to-volume ratio (SA:V) is a crucial parameter for NPs activity, 30 nm Au@Pt and ultrasmall 2 nm PtNPs were investigated, primarily to show the differences in platinum reactivity in different ways of its immobilization in NPs. Our additional purpose was to find out about the possibility to release Pt from nanoparticles, especially from Au@Pt, as a concept to deliver ^195m^Pt, radionuclide for Auger therapy, which is planned to be applied for these nanomaterials.

We performed multidimensional analyses to evaluate interactions between cancer cell lines and different types of Pt nanoparticles. The cytotoxic effect was observed for up to 72 h, with particular attention to the ROS production, nuclear uptake study and apoptosis induction. Finally, by employing 3D tumor spheroid models, we assessed the ability of Au@Pt and PtNP to penetrate tissue and still deliver cytotoxic therapy. For HER2+ breast and ovarian cancers, trastuzumab-targeted bioconjugates were used, whereas the experiments with only polymer-stabilized Au@Pt and PtNPs were performed in HepG2 cells, commonly known to be a cancer cell line with a high degree of oxidative stress. HepG2 cells are highly heterogenic, and any targeting-available receptors on the cell membrane which could be applied were identified.

## 2. Results and Discussion

### 2.1. Characterization of Investigated Platinum Particles

Here, we speculate that platinum-containing nanoparticles are good candidates to become an anticancer agent in highly oxidized environments. As ultrasmall NPs have a high surface-to-volume ratio, their catalytic properties should be significantly enhanced in comparison to large-diameter nanomaterials. This is due to the increased number of Pt atoms on the particles’ external surface, which is correlated with radius (Table 1). On the other hand, immobilization of platinum on gold nanoparticle core gives an interesting possibility for further application of radioactive Auger electrons emitter ^195m^Pt for combined chemo-radiotherapy. Due to the very short tissue range, Pt deposition on AuNPs seems to be more promising from a radiotherapeutics perspective, which is planned to be applied for these nanomaterials.

Regarding a dependence between physicochemical properties of Pt particles and their biological function, we assumed that one PtNP with a diameter of around 2 nm has, on its surface, 161 atoms of Pt that correspond to 61% of all atoms located on the NPs’ surface. In contrast to PtNPs, a lower percentage of platinum atoms (only 8.9%) are present on the outer Au@Pt shell, which may suggest lower reactivity of these nanostructures when compared to 2 nm PtNPs. One of the hypothesized mechanisms of action for Pt interaction with H_2_O_2_ assumes that during reaction with hydrogen peroxide, a Pt^2+^ cation can be released from the NP [5]. An oxidized form of Pt has high reactivity; for instance, it easily penetrates the nuclear envelope and can directly intercalate the DNA structure [6]. This may consequently lead to an improved cytotoxicity of Pt and its increased therapeutic efficacy. Moreover, due to the small diameter, only 2 nm nanoparticles can technically reach cellular DNA by penetration of the nuclear pore complex, which is permeable for molecules with molecular weight in a range between 30 and 60 kDa and up to 5 nm in diameter [7].

### 2.2. Uptake of Nanoparticles in HepG2 and SKOV-3 Cells

Internalization to the cytoplasm is crucial for effective platinum interactions with hydrogen peroxide. For cells-internalization assay in HepG2 cells, polymer-stabilized nanoparticles were used, while for SKOV-3 nanoparticles bioconjugates with monoclonal antibody (mAb), trastuzumab, which is widely used for the therapy of HER2-positive cancers, was used. Bioconjugation of nanoparticles with targeting vector was carried out in order to enhance the binding and internalization ratio to SKOV-3 cells (Supplementary Information, Figures S2 and S3). As calculated with radiometric assays, there were around 22 and 2 trastuzumab molecules per one Au@Pt and PtNP conjugated, respectively (Supplementary Information).

**Figure 3:**
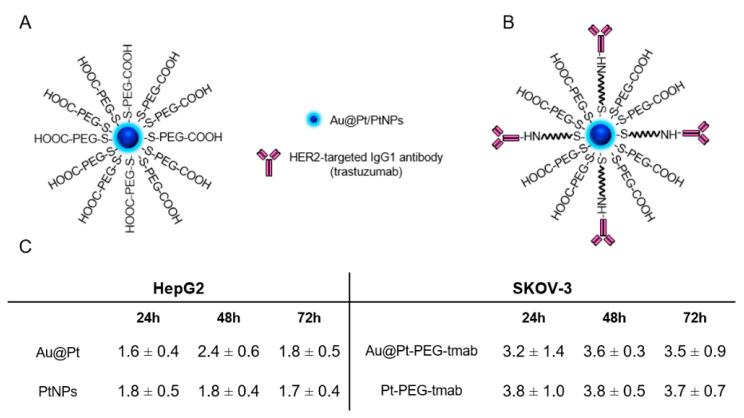
Schematic-structures illustrations of PEG-stabilized nanoparticles (**A**) or bioconjugates with trastuzumab (**B**); internalization [%] of Au@Pt-PEG/PtNPs-PEG in HepG2 cells and Au@Pt-PEG-trastuzumab/PtNPs-PEG-trastuzumab in SKOV-3 cells (n = 3, for three independent experiments) (**C**).

Intracellular content of both types of nanoparticles was in the range of 1.6–2.4% and 3.2–3.8% for HepG2 and SKOV-3 cells, respectively (Figure 3C). PEG-ylated nanoparticles were able to penetrate only HepG2 cells, as SKOV-3 cell membrane was impermeable for conjugates without an antibody. Any meaningful differences between Au@Pts’ and PtNPs’ uptake in HepG2 and SKOV-3 cells were found. During incubation, no decrease in cellular content of the tested compounds was observed; thus, it was confirmed that nanoparticles are retained in cytosol for at least 72 h. Long-term retention of both NP types displays their promising therapeutic efficacy, especially for inducing significant levels of reactive oxygen species.

### 2.3. Cytotoxicity Evaluation after Incubation of Cancer Cells with the Different Pt Particles and Their Bioconjugates

**Figure 4:**
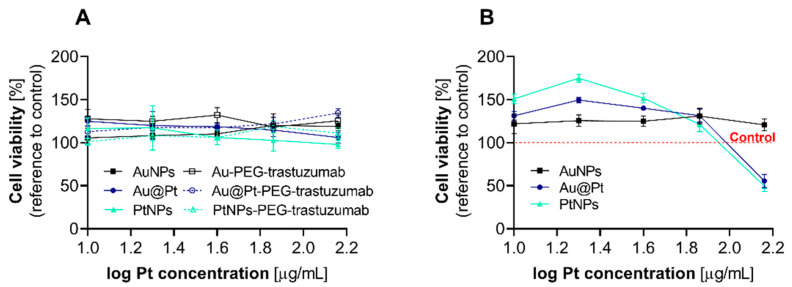
Cell viability after 72 h incubation of SKOV-3 (**A**) and HepG2 (**B**) cells with Au@Pt, PtNPs, their bioconjugates and AuNPs (as control). As presented, any impact of tested compounds on the ovarian cancer cells’ proliferation was observed. In contrast, a significant decrease in cell viability was observed in HepG2 cells treated with platinum-containing nanoparticles, and any response was found for AuNPs.

Cellular viability investigations of Au@Pt and PtNPs were performed with SKOV-3 and HepG2 cells. Both cell lines are known to be in vitro examples with increased intracellular hydrogen peroxide production [8,9]. We chose MTS (3-[4,5,dimethylthiazol-2-yl]-5-[3-carboxymethoxy-phenyl]-2-[4-sulfophenyl]-2H-tetrazolium, inner salt) assay for an assessment of the toxicity of NPs with the reference to the oxidative redox imbalance caused by the particles [10]. Neither Au@Pt, PtNPs nor their bioconjugates with trastuzumab inhibited the cell proliferation of the SKOV-3 cell line (Figure 4A). Both PtNPs and Au@Pt consistently exerted the highest cytotoxicity (49.7% and 55.7%, respectively) if hepatoma cells were treated with 145 µg/mL of Pt (log 2.16). For doses lower than 145 µg/mL, no impact on cell viability was observed (Figure 4B). To clarify whether the main source of cytotoxicity in the analyzed particles comes from the presence of Pt atoms, we introduced 30 nm gold nanoparticles (AuNPs) without a platinum layer. Indeed, no changes of HepG2 viability were observed during this treatment, which confirmed the role of platinum in the NP-mediated cytotoxicity. The above-described changes induced by Pt-containing nanoparticles were observed in a time-dependent manner, with the highest toxicity index after 72 h of incubation (Supplementary Information, Figure S4).

In general, platinum-induced cytotoxicity can be observed at various concentrations, although this is highly dependent on size, NP surface charge, their chemical modifications and cell lines used for evaluation. Another critical role of different particles’ toxicity is the possibility of their absorption by serum proteins [11]. This phenomenon, known as the “corona effect”, significantly decreases the interactions between NPs and cancer cells and reduces toxicity [12]. The variable sensitivity of tumor cells to platinum nanoparticles has been described many times [13]. With regards to our previous study, we found that PtNPs exhibit cytotoxic properties in concentrations between 50 and 400 µg/mL [14]. Almeer et al. [15] observed similar toxicity during incubation of HEK293 cells with 360 µg/mL PtNPs (~24 nm). Conversely, Mironava et al. [16] calculated the IC50 parameter of Pt to be between 51 and 124 µg/mL when folate-functionalized nanoparticles (dimeter range 2–15 nm) were used against breast cancer cells, which clearly corresponds to the data presented herein.

### 2.4. The Effect of PtNPs, Au@Pt and Their Bioconjugates on Redox Homeostasis in Cancer Cell Lines

A variety of NPs have been shown to alter cellular oxidative homeostasis either by overproduction of ROS or by depleting the cellular reserve of reduced glutathione (GSH) [17]. Especially regarding the nanomedicine of cancer, a redox imbalance is one of the crucial factors affecting cell signaling and tumor progression. Therefore, we compared the ROS level and GSH concentration in a corresponding number of untreated HepG2 and SKOV-3 cells and referenced according to the BCA (bicinchoninic acid) assay. The obtained results demonstrated a negligible increase in ROS level at SKOV-3, while in hepatic cells signal, it was more than twice higher. This corresponds with GSH and DCFDAH_2_ oxidation levels in untreated cells, which indicates higher antioxidative resistance of HER2+ cells and their lower oxidative status when compared to HCC (Supplementary Information, Figure S5).

**Figure 5:**
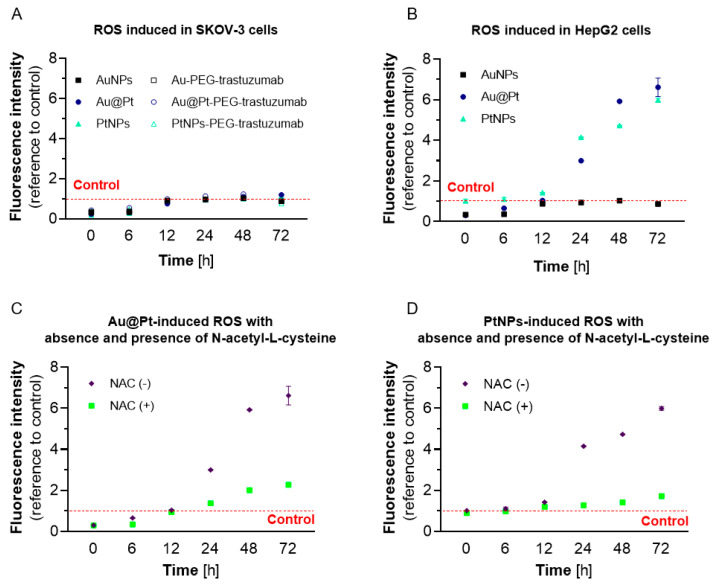
Free radical production in SKOV-3 and HepG2 cells: any ROS overproduction was found after treatment SKOV-3 with Au@Pt, PtNPs and AuNPs (as control) and their bioconjugates (**A**); in contrast, significant ROS concentration was induced from Au@Pt and PtNPs in HepG2 cells (**B**). As expected, decrease in ROS fluorescence intensity was observed with preincubation with NAC, which was used as protectant (**C**,**D**). The concentration of Pt in these nanoparticles during experiments was always 145 µg/mL.

Different physiology and cytotoxicity profiles do not exclude the possibility of ROS formation in the cell lines analyzed. Therefore, we aimed to clarify the level of redox stress induced by Au@Pt and PtNPs in HepG2 and SKOV-3 cells. ROS induction was measured in a time-dependent manner (Figure 5). To confirm that free-radical production is a consequence of treatment with the experimental particles, we performed experiments using a pretreatment with the ROS scavenger N-acetyl-L-cysteine (NAC).

No ROS formation was induced in SKOV-3 cells after treatment with Au@Pt/PtNPs particles or their bioconjugates with trastuzumab (Figure 5A). These results are in agreement with MTS assay, wherein the investigated cell viability was not significantly altered. In contrast, Pt-treated HepG2 cells displayed increased ROS over time and reached a maximum concentration at 72 h of incubation with Au@Pt, as well as PtNPs (Figure 5B). At this time, the level of ROS was increased by approximately 6-fold (PtNPs; *p* < 0.0001) and 6.6-fold (Au@Pt; *p* < 0.0001) when compared to the untreated control cells. AuNPs did not affect ROS-related fluorescence intensity, and thus confirms that Pt plays an essential role in oxidative-stress induction and enhanced cytotoxicity. During simultaneous treatment of cells with Pt particles and NAC (Figure 5C,D), we observed a significant decrease in DCFDAH_2_ fluorescence intensity during all time points. This confirms that an increase in recorded signal is a result of ROS-induction following NP application to the HepG2 and SKOV-3 cell cultures.

Interestingly, PtNP-induced ROS was more effective after just 12 h of incubation and reached 1.4-fold growth increase compared to the control cells. This observation may be related to the higher reactivity of 2 nm NPs that comes from their significant surface-to-volume ratio. Our results are consistent with data previously published by Almeer et al. [15], whose evaluation of pro-oxidative activity of PtNPs (<100 nm) was performed in HEK293 cells. They observed a cytotoxicity-related ROS overproduction (122% of control) and a decrease in GSH levels. Nonetheless, the pro-oxidative activity of tested compounds was inferior when compared to our study, likely due to the different NP sizes and cell lines used.

### 2.5. Effects of Oxidative Stress in HepG2 Cells

**Figure 6:**
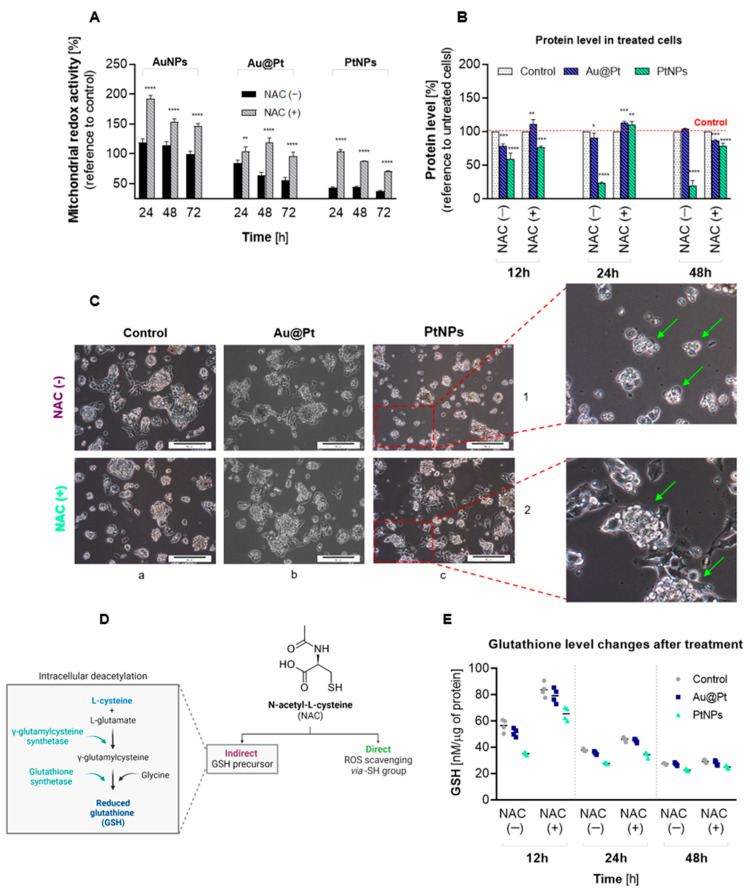
Redox imbalance of HepG2 cells treated with Au@Pt and PtNPs: Cytotoxic effect of Au@Pt and PtNPs preatreated with NAC in a time-dependent manner (**A**); the changes of protein level in cells treated with NPs (**B**); micoroscopy images of morphological alterations after HepG2 cells’ treatment with Au@Pt and PtNPs (scale bare on presented images corresponds to 50 μm, images were done with 10× magnification) (**C**); mechanism of NAC antioxidant activity inside the mmamlian cell (**D**); GSH level alterations (**E**). As presented, cell viability reduction was related to GSH and protein-level reduction as well as affected in the significant morphological changes in HepG2 cells. Due to the significant toxicity of the NPs, all calculations were made with reference to the intracellular protein content. Statistical analyses were performed with regard to control cells (**A**,**B**). The results are presented as mean ± SD. *p*-values are presented as: (*) *p* ≤ 0.05, (**) *p* ≤ 0.01, (***) *p* ≤ 0.001 and (****) *p* ≤ 0.0001.

Taking into account that Au@Pt and PtNPs do not induce any cytotoxicity or ROS overproduction, our further studies were concerned with HepG2 cells. Having shown that ROS formation was inhibited in the presence of NAC, we asked whether this antioxidant has a protective effect on HepG2 viability during PtNPs and Au@Pt treatment. NAC strongly inhibited toxicity induced by Au@Pt as well as PtNPs for all investigated time points (Figure 6A). NP treatment was also reflected at the protein expression level (Figure 6B). Firstly, we noticed a significant decrease in the amount of total protein in cells following 12 h incubation, reaching 79% (*p* < 0.001) and 59.1% (*p* < 0.0001) in Au@Pt or PtNPs-treated cells, respectively. A similar difference between core-shell and 2 nm NPs was observed following a 24 h or 48 h incubation. In contrast to Au@Pt, PtNPs induced a long-term reduction of cellular protein content that reached a minimum (19.2%) if particles were present in a cell culture for up to 72 h. The fact that NAC increased metabolic activity of HepG2 cells treated with NPs was also confirmed by cellular morphology (Figure 6C). A preincubation of cells with NAC significantly increased the number of cells after treatment with NPs. Interestingly, there were substantial morphological differences between Au@Pt-treated and PtNPs-treated cells. Core-shell NPs (Figure 6C, panel 1b) affected a reduction in cell numbers and a slight loss of cell-cluster integrity, visualized by the separation of single cells outside of clusters. Other changes were noted in cells treated with 2 nm nanoparticles (Figure 6C, panel 1c), including not only a significant decrease in cell numbers but also cell shrinkage, loss of cluster integrity and changes to cellular shape.

Since we noted that NAC diminished PtNPs/Au@Pt-mediated ROS production and cytotoxicity, we next measured the alteration of intracellular GSH, the main nonenzymatic cellular antioxidant. Reduced GSH plays an essential role in the protection against free radicals. NAC is a precursor for GSH synthesis in cells and can act as an antioxidant directly by the ROS-scavenging via –SH groups or indirectly (Figure 6D). In our study, HepG2 cells were incubated with Au@Pt and PtNPs (145 µg/mL) at three time periods, being 12 h, 24 h and 48 h. GSH changes were analyzed in comparison to samples pretreated with NAC. The first GSH alterations were noticed after 12 h of incubation with examined PtNPs (Figure 6E). Ultrasmall nanoparticles (2 nm) induced a higher (1.60-fold) decrease in GSH, whereas Au@Pt diminished GSH concentration 1.13-fold. As expected, GSH content increased in cells preincubated with NAC but did not achieve the level of the untreated control cells. Interestingly, HepG2 cells treated with Au@Pt for 24 h and 48 h were able to restore the GSH level to a similar concentration as in control cells. These data are in contrast with the results obtained for HCC cultures treated with ultrasmall PtNPs. A substantial depletion of GSH content in Pt-treated cells after 24 and 48 h reached 28 nM/µg protein and 21 nM/µg protein, respectively, with reference to the untreated control cells (39 nM/µg protein for 24 h and 31 nM/µg protein for 48 h). A decrease in the GSH level after pro-oxidative Pt-containing NPs was also observed in previously published research with different cell lines [18,19,20]. It was concluded that GSH depletion is highly dependent on the size of PtNPs, wherein usually smaller nanoparticles (<20 nm) were able to induce a more significant reduction of GSH level. Research conducted by Li et al. [21] demonstrated that Pt applied in complexed form (Se-Pt) also leads to depletion of GSH level in HepG2 cells and induces oxidative stress, highlighting that Pt can act as a pro-oxidant either as a metal or ionic complex.

### 2.6. Nuclear Uptake and Cell Cycle Arrest

**Figure 7:**
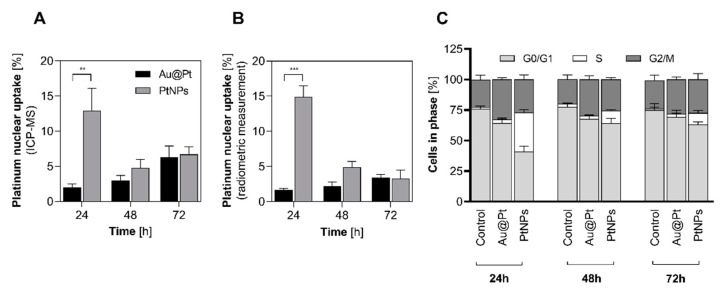
Nuclear platinum uptake and its consequences for cell cycle distribution. Platinum nuclear uptake: (**A**) ICP-MS, (**B**) radiometric measurement. Significantly higher uptake after 24 h was noticed for 2 nm PtNPs when compared to 30 nm Au@Pt (*p* < 0.01). The blockage of HepG2 cell cycle induced with Au@Pt and PtNPs—percentage distribution of G_0_/G_1_:S:G_2_/M phases in control cells and treated with nanoparticles (**C**). The results are presented as mean ± SD. *P*-values are presented as: (**) *p* ≤ 0.01, (***) *p* ≤ 0.001.

Platinum nanoparticles have diverse behavior in highly oxidized environments; thus, we examined whether Pt can be released from the investigated particles and form a soluble fraction of Pt^2+^ cation in a cellular environment. To answer this question, we estimated the percentage of internalized Pt into nucleus from cell cytosol. To examine internalization efficiency, we isolated cell nuclei fractions from HepG2 cells treated with Au@Pt and PtNPs (Figure 7A,B) and measured intracellular Pt concentration after 24 h, 48 h and 72 h of incubation. Due to the high sensitivity of this measurement, despite ICP-MS evaluation of Pt content in cell nucleus (Figure 7A), we conducted a cross-reference investigation with Au@[^197^Pt]Pt and [^197^Pt]PtNPs with subsequent radiometric assay (Figure 7B). Although long-term retention of both NP types displays their promising therapeutic efficacy, our results for Au@Pt internalization to nucleus exclude the hypothesis that Pt^2+^ cation can be released from NPs. Why did we want to validate this hypothesis? Firstly, only trace amounts of Pt appeared in the nuclei fraction (Figure 7A,B, black bars), although the Au:Pt ratio in the nuclei fraction was unchanged. In general, the presence of trace amounts of gold is probably due to the aftermath of contamination of the nucleus with incompletely washed Au@Pt during the isolation procedure. We assumed that if a Pt cation would dissociate from its NP, the 1:1 ratio of Au:Pt obtained during synthesis should decrease (Supplementary Information, Figure S7). Thus, we excluded a theory presented by Shoshan et al. [5] consisting of forming Pt^2+^ cation in the case of Au@Pt. In contrast, we observed quite different results for PtNPs. It was discovered that around 13–15% of internalized Pt was retained in the nucleus after 24 h of incubation (Figure 7A,B, grey bars), with minor differences reported for both of techniques. This is much less than data reported by Shoshan, but such a high uptake (*p* < 0.01) is not accidental. Based on this discrepancy, it is tempting to speculate that in the case of ultrasmall PtNPs, we cannot unambiguously confirm or exclude a formation of the Pt cation after intracellular particle uptake. Moreover, the size of PtNPs facilitates nuclear-envelope penetration via nuclear pore channels which are around 5 nm diameter [22]. Therefore, nuclear location of PtNPs can be a consequence of two different biological routes, but taking into account lack of Pt dissociation from Au@Pt, we hypothesize that the nuclear-envelope penetration of metallic nanoparticles is more likely.

Since we observed that PtNPs may successfully locate in the cell nucleus, we asked whether modifications of the cellular genome induced by examined NPs followed the alterations in cell-cycle phases. Previously published literature demonstrated that NPs with noble metals may provoke cell cycle arrest in G_0_/G_1_ and G_2_/M phases [14,23]. Indeed, DNA staining with PI revealed that both Pt-containing NPs altered cell cycle distribution in a variety of ways (Figure 7C and Supplementary Information, Figure S8). Conversely, we noted that neither Au@Pt nor PtNPs blocked HepG2 cells in G_2_/M phase, an observation consistent with Li et al. [24], who revealed that porous PtNPs themselves had no effect on G_2_/M arrest in the NCIH460 cell line but significantly enhanced radiation-induced G_2_/M arrest after a combined treatment with 50 μg/mL porous PtNPs and X-ray irradiation. A surprisingly high percentage of S phase cells (31.9%) was noted in HepG2 cells treated with PtNPs for 24 h. During prolonged incubation for up to 48 h with ultrasmall PtNPs, the distribution of S-phase cells reduced to 9.6%, while the G_0_/G_1_ phase increased and reached 27.3% after 72 h of continuous NP treatment. This agrees with the previously described reduction of intercellular protein content. During the G_1_ phase, cellular supplies of proteins increase to prepare the cell for further DNA replication. When the G_1_/S checkpoint is reached and passed, the cell progresses to the S-phase, and the previously synthesized proteins are widely used. This can directly explain differences between cell cycle distribution, protein synthesis level and cytotoxicity of Au@Pt and PtNPs. Moreover, this also confirms that according to ICP-MS measurements, Au@Pt does not dissolve to release platinum cation, which can internalize into the nucleus.

A block of the G_0_/G_1_ phase suggests oxidative-stress induction in the cell’s genome [25,26], whereas an accumulation of cells in S-phase is typical for PtNPs with small diameter that show proapoptotic properties [27]. These data support our thesis that Pt successfully penetrated the nuclear envelope. Interestingly, the high percentage of cells in S-phase after 24 h of incubation with PtNPs correlates with the data that demonstrate almost 13% of Pt coming from 2 nm PtNPs was found inside the nucleus. This similarity was discussed by Wang et al. [28], who highlighted that cis-platinum, a well-known DNA-intercalator, causes S-phase arrest in HepG2 cells. They also noted that S-phase arrest should occur at the early time points when cells activate different prosurvival pathways of the cellular stress response. This may be applicable to our research, where S-phase distribution came to the control level after 48 and 72 h of treatment with PtNPs.

According to these results, we hypothesize that inhibition of protein synthesis in the presence of PtNPs in HepG2 cells in vitro may be related to its intranuclear location and S-phase cell cycle arrest. This can directly explain differences between cell cycle distribution, protein synthesis level and cytotoxicity of Au@Pt and PtNPs. Ultrasmall NPs induced S-phase arrest, during which protein synthesis reached a very low level [29]. This drop in cellular protein content was totally abolished by preincubation with NAC.

### 2.7. The Induction of Apoptosis in HepG2 Cells Treated with PtNPs and Au@Pt

**Figure 8:**
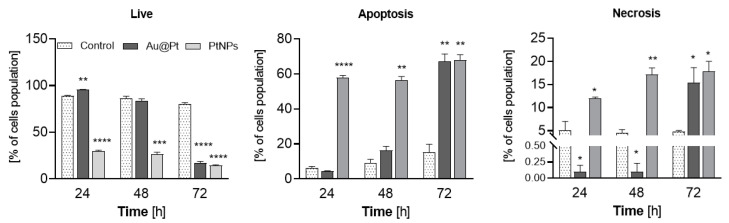
Annexin V assay in HepG2 cells after platinum nanoparticles treatment: live, apoptotic and necrotic cells’ distribution changes over time—a different ratio of apoptotic and necrotic cells was found especially after 24 and 48 h, while 72 h toxicity profile was similiar for Au@Pt and PtNPs. The results are presented as mean ± SD. *P*-values are presented as: (*) *p* ≤ 0.05, (**) *p* ≤ 0.01, (***) *p* ≤ 0.001 and (****) *p* ≤ 0.0001.

We next aimed to identify the mode of programed cell death induced by nanoparticle variants. There are many molecules, such as free radicals, that act as second messengers and contribute to the induction of apoptosis [30]. Since we observed a marked, time-dependent increase in ROS production, we asked whether redox stress triggered by Au@Pt and PtNPs refers to the particle-dependent, apoptotic changes in HCC. The typical, molecular hallmarks of apoptosis, such as chromatin condensation and apoptotic body formation, have been previously confirmed with confocal imaging of HCCs after treatment with Au@Pt [31]. To extend that research, here, we employed Annexin-V assay. The three cell populations, live, apoptotic and necrotic, were distinguished during flow cytometry measurements (Figure 8 and Supplementary Information, Figure S6), and a time-dependent difference between the NPs was determined. These data are in agreement with our cytotoxicity studies performed using the MTS assay. For instance, a short treatment time with core-shell NPs at a Pt concentration of 145 µg/mL triggered no toxic effects, and neither apoptosis nor necrosis in HCC cultured in vitro were observed. However, a prolonged treatment of HepG2 cells with Au@Pt particles for up to 72 h induced the highest statistically significant fraction of apoptotic (Annexin V-FITC+/PI+) and necrotic (Annexin V-FITC-/PI+) cells. At this time point, only 17.1% of cells with full cell membrane integrity remained (*p* < 0.0001).

In contrast, toxicity-inducing ultrasmall PtNPs triggered proapoptotic changes in HepG2 much earlier. We observed that after 24 h, the fraction of live cells was significantly (*p* < 0.0001) reduced to 30%. Furthermore, this decrease in cell viability was 2-fold higher if HepG2 cells were treated with PtNPs for up to 72 h. In parallel, if HCC cells were treated with ultrasmall PtNPs, a gradual increase in the percentage of Annexin-V binding cells appeared and reached 58% (*p* < 0.01) and 67.8% (*p* < 0.01) after 48 h and 72 h of incubation, respectively. A greater fraction of apoptotic cells corresponded with a substantial increase in PI-positive cells (from 12% at 24 h of incubation to 17.4% after 72 h; *p* < 0.05). We did not observe this phenomenon earlier for Au@Pt-treated cells, implying a difference between toxicity mechanism of the two PtNPs.

We noted a high percentage of cells that were stained with propidium iodide and FITC. These data suggest that treatment of HepG2 cells with Au@Pt and PtNPs caused a loss of cell membrane integrity and triggered DNA cleavage, which was confirmed with PI staining.

### 2.8. The Influence of PtNPs and Au@Pt on Three-Dimensional Spheroid Cell Cultures

The application of Pt in NPs has several advantages, including a better biodistribution of the anticancer agent, mostly due to an enhanced permeability and retention (EPR) effect. However, for a comprehensive determination of newly designed Pt nanoparticles as potential anticancer agents, it was important to apply not only 2D cell cultures, but also 3D tumor models. Employing 3D models to this area of research provides more trustworthy data from a model cancer tissue. Moreover, with three-dimensional spheroids, the dependence on cell-to-cell interactions and a development of multidrug resistance phenomenon can be more adequately observed. Especially with regards to NPs with a larger spherical size than conventional chemotherapeutics, the experiments with 3D tumor models are crucial [32].

**Figure 9:**
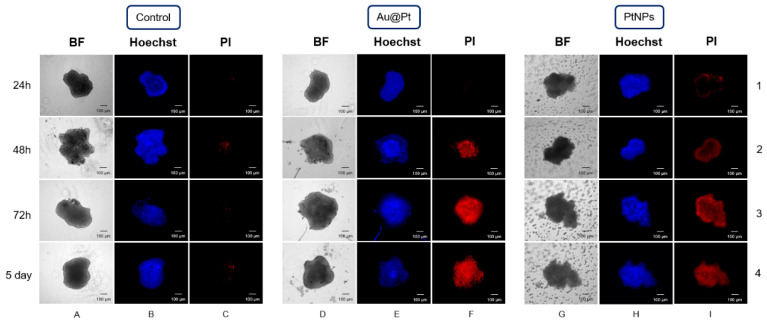
Fluorescent imagaing of 3D HepG2. Columns (**A**,**D**,**G**)—bright field; columns (**B**,**E**,**H**)—Hoechst 33258-stained cells (blue); columns (**C**,**F**,**I**)—propidium iodide-stained cells (red).

Here, an evaluation of cytotoxic activity of Au@Pt and PtNPs (C_Pt_ = 145 µg/mL) was performed with nonquantitative fluorescence imaging using Hoechst 33258 and PI. Spheroids were cultured for 5–7 days before treatment or until the diameter reached ~250–350 µm. This avoids any potential variability in homogeneity, and oxygen and nutrient availability, which can facilitate the formation of a central necrotic core [33]. During each experimental day, we compared the intensity of signals that came from fluorochromes in treated versus untreated control cells. The images were collected after 24 h, 48 h, 72 h and 120 h (5 days) of continuous incubation with Au@Pt and PtNPs, respectively (Figure 9).

As we expected, HepG2 3D spheroids revealed an irregular shape similar to the unusual morphology and growth pattern of HCC in 2D cultures. Both types of tested NPs did not induce any (Au@Pt) or very weak (PtNPs) toxicity after 24 h (Figure 9, 1F and 1I). This is a valuable observation, especially regarding 2 nm PtNPs, which showed high cytotoxicity after 24 h treatment during MTS assay and flow cytometry measurements. However, if the incubation with Au@Pt and PtNPs was prolonged from 48 h to 120 h, we noticed an increasing PI signal. This is in accordance with our expectations that both NPs can penetrate tumor tissue. However, even if spheroids showed no disintegration or loss of integrity after 5 days of treatment (Figure 9, 4D and 4G), a strong PI signal was still easy to distinguish.

## 3. Materials and Methods

### 3.1. Uptake of Nanoparticles in HepG2 and SKOV-3 Cells

To evaluate the availability of PtNPs to successfully penetrate the cell membrane and nuclear envelope, the Nuclei EZ Preparation Kit (Merck & Co., Inc., Kenilworth, NJ, USA) was used. For this purpose, 1.5 × 10^7^ HepG2 cells were seeded into 100 mm petri dishes for 24 h incubation Then, the media were removed, and cells were treated with Au@Pt, PtNPs or their bioconjugates (doses corresponding to 5 nM of bioconjugate) and were added in 8 mL of fresh medium for a 24 h, 48 h and 72 h incubation. The whole procedure was performed in agreement with the manufacturer’s protocol. Cells were washed with ice-cold PBS and harvested using a cell scraper following incubation with Nuclei EZ Lysis buffer. Next, lysates were centrifuged (4 °C, 500× *g*, 5 min). This step was repeated twice, and the supernatant was collected as the cytoplasmic fraction. The samples were then analyzed via the inductively coupled plasma mass spectrometry (ICP-MS) technique (Elan DRC II, Perkin Elmer, Waltham, MA, USA).

### 3.2. Cytotoxicity Studies

Cytotoxicity studies were performed in two different variations. At first, the cytotoxicity of Au@Pt and PtNPs was evaluated in SKOV-3 and HepG2 cells with different concentrations of conjugates (Au@Pt/PtNPs-PEG-COOH-HepG2 and SKOV-3) and bioconjugates (Au@Pt/PtNPs-PEG-trastuzumab–SKOV-3). The second experiment included only HepG2 cells with 60 min preincubation with NAC as an ROS scavenger. For both experiments, 3–4.5 × 10^3^ cells were seeded into 96-well plates and incubated overnight (37 °C, 5% CO_2_). Subsequently, the media were replaced with tested compounds in a growing medium. Cells were incubated with nanoparticles for 24 h, 48 h and 72 h. Following incubation, the media were removed and stored, and fresh media were added. Finally, 20 µL of MTS-CellTiter96^®^ AQueous One Solution Reagent (Promega, Madison, MI, USA) was added, and absorbance was measured at 490 nm to calculate the % of metabolically active cells.

### 3.3. Reactive Oxygen Species (ROS)

Fluorescent CM-H_2_DCFDA was used as a specific probe for ROS. This probe uses chloromethyl groups that enhance cytosol retention via covalent bond with thiols in cellular structures [34]. Following internalization, the probe is deacetylated in the cytosol. This nonfluorescent intermediate can then be oxidized via ROS, leading to the formation of a fluorescent product. Experiments were performed as reported elsewhere [35]. In brief, HepG2 cells (1–2 × 10^4^) were seeded into 96-well black plates with a transparent bottom for overnight incubation. The next day, the cells were washed once with PBS, and a 45 min preincubation with probe in growing medium w/o phenol red was completed. Subsequently, cells were washed with PBS once, and a 60 min incubation with NAC was performed to mop up any remaining ROS. Finally, cells were treated with appropriate concentrations of Au@Pt-PEG-COOH, PtNPs-PEG-COOH and hydrogen peroxide as the positive control. Fluorescence measurements (λ_ex_ = 495 nm; λ_em_ = 535 nm) were performed with a TRISTAR LB 231 Multimode Reader (Berthold Technologies GmbH & Co. KG, Bad Wildbad, Germany). For longer incubations (t > 24 h), cells were incubated with a probe directly prior to the readout instead of at the beginning of experiment. To avoid errors in the measurement of fluorescence intensity caused by feasible cell detachment and resulting in a decrease in the cell number in the samples due to the drug treatment, the MTS assay readouts were performed afterwards. Finally, the data presented in Figure 5 were standardized to the number of viable cells assessed by cell viability assay applied during this study.

### 3.4. Glutathione Level Changes

We analyzed changes in reduced glutathione (GSH) in cells treated with Au@Pt and PtNPs with fluorescent measurements (λ_ex_ = 355 nm; λ_em_ = 460 nm) according to the previously described literature [36]. Briefly, 6 × 10^5^ of HepG2 cells were seeded into 6-well plates and incubated overnight. Next, cells were washed with either PBS or a growing medium; then, a 45 min preincubation with NAC was performed. NPs at a concentration of 145 µg/mL were added to wells and incubated for 12 h, 24 h and 48 h. During each experiment, further steps were made as follows: after trypsinization with 0.05% trypsin EDTA solution, cells were centrifuged (300× *g*, 5 min, 4 °C) and pellets were dispersed in lysis buffer (NaCl, Na_2_HPO_4_ and EDTA; pH ~ 7.40) supplemented with Triton X-100 on ice for 10 min. Lysates were diluted 1:1 with assay buffer (TCA, DTPA, HCl)/ascorbic acid (1:1), and after 30 min of protein precipitation, cells were centrifuged at 13,000 rpm (10 min, 4 °C). Supernatant was added into 96-well black plates. For this assay, GSH reaction with o-phthaldialdehyde with formation of hemithioacetal group was used. This group is reactive toward primary amines, which leads to the formation of the fluorescent isindole complex. In these experiments, TCA-redox quenching buffer (TCA-RQB) and potassium phosphate buffer were added to each well. In half of the wells, N-ethylmaleimide was added for GSH derivatization, while the second part was mixed with a reducing-quenching buffer. In the last step, plates were incubated for 5 min at RT, and OPA was added. For the calibration curve, GSH in concentrations of 0–0.5 mM was used. All calculations were based on the calibration curve made with GSH. The fluorescence measurement was performed with Fluoroskan Ascent FL microplate reader (Thermo Fischer Scientific, Waltham, MA, USA).

### 3.5. Protein Level Changes

To correctly calculate changes to the GSH level, total protein in both control and treated cells was measured using a Pierce™ BCA Protein Assay Kit (λ = 660 nm) with a BSA calibration curve. Briefly, after incubating cells with lysis buffer/triton X-100, small portions of lysates were pipetted directly into 96-well plates, and Pierce reagent was added. The plates were then incubated for 5 min in RT, and absorbance was measured with a Microplate UV–Vis Spectrophotometer (Biotek Eon, Waltham, MA, USA). In parallel, cell morphology alterations were analyzed following 24 h of exposure to NPs. Images were acquired using an inverted microscope (Olympus IX70, Japan) equipped with a 20× objective and a Digital Sight camera (Olympus, Tokyo, Japan).

### 3.6. Nuclear Uptake and Cell Cycle Arrest

For this experiment, pellets obtained during cell fractionation were used. Pellets containing separated nuclei were dispersed in 200 µL of storage buffer, and the samples were stored at −20 °C. Pt concentration in the cytosol and cell nucleus was determined using inductively coupled plasma mass spectrometry (ICP-MS) or radiometric assay by Wizard^2^ Detector Gamma Counter (Perkin Elmer, Waltham, MA, USA).

For cell cycle analyses, 8 × 10^5^ HepG2 cells were seeded into 6-well plates. After an overnight incubation, 145 µg/mL of Au@Pt-PtNPs-PEG-COOH was added for 24 h, 48 h and 72 h incubations. Once the experimental time was up, cells were washed with ice-cold PBS and removed from the plate with 0.05% trypsin EDTA sol. C. During the cell cycle analysis, a similar procedure was applied, with an additional step. After trypsynization and centrifugation, cells were fixed with ice-cold 70% EtOH and stored for at least 1.5 h in −20 °C. EtOH was then removed by washing with PBS and centrifugation. Cell pellets were resuspended in PBS and propidium iodide and RNAse was added for 45 min (37 °C, 5% CO_2_). Samples were analyzed with BD FACSCelesta™ and BD FACSDiva Software, v.8.0.1.1 (BD Bioscience, Franklin Lakes, NJ, USA). ModFit LT™ 5.0 software (Verity Software House Topsham, ME, USA) was additionally used to analyze cell cycle arrest.

### 3.7. Flow Cytometry–Apoptosis

During the apoptosis analysis, a similar procedure was applied, with an additional step. The cells were centrifuged, and pellets were resuspended in 100 µL of Annexin-V 1× Binding Buffer. Next, Annexin-V FITC and propidium iodide were added and incubated in 37 °C in darkness for staining. Finally, Annexin-V Binding Buffer 1× was added.

### 3.8. Spheroids

Tumor spheroid models were cultured with a similar procedure as that used previously [37]. Briefly, 1 × 10^3^ HepG2 cells in 200 µL of growing medium were seeded into 96-well U-bottom ultralow adherent plates (Corning^®^, Corning, NY, USA) seven days before the experiment. During incubation, 100 µL of medium was replaced every two days. After spheroids had expanded to approximately 250–350 µm diameter, Au@Pt and PtNPs conjugates were added. Before imaging, spheroids were washed 3–5 times with PBS. For staining, Hoechst 33258 (permeable for live and dead cells) and propidium iodide (permeable only for dead cells) were used in accordance with manufacturers guidelines. Fluorescent imaging was performed with a ZEISS Axio Vert.A1 Microscope and ZEN 2.1 software (Zeiss, Jena, Germany).

### 3.9. Statistical Analysis

For statistical analysis, one-way ANOVA and Student’s *t*-tests were used in GraphPad Prism v.8 Software (GraphPad Software, San Diego, CA, USA). The results are presented as mean ± SD. *P*-values are presented as: (*) *p* ≤ 0.05, (**) *p* ≤ 0.01, (***) *p* ≤ 0.001 and (****) *p* ≤ 0.0001.

## 4. Conclusions

To the best of our knowledge, the current work represents the first time a comparison of Au@Pt and PtNPs has been performed regarding their cytotoxicity and pro-oxidative mechanism. To optimize their further application, we need to understand their mechanism of action on cancer cells. The experiments related to the biological activity of PEGylated 30 nm Au@Pt and 2 nm Pt nanoparticles show significant differences between the two types of NPs. Both NPs displayed high toxicity toward HCC cells and had a neutral effect on the ovarian SKOV-3 cell line. No cytotoxic effect was observed if either of the Pt-based bioconjugates of NP/trastuzumab were used. The high concentration of H_2_O_2_ in HepG2 improved the lethal effect of NPs that corresponds to their apoptotic and genotoxic properties estimated during flow cytometry. Simultaneously, we demonstrated that Au@Pt nanoparticles’ effect was noticeably less toxic. Based on our results, we conclude that both Au@Pt and PtNPs, following internalization, act as pro-oxidants due to different interactions with hydrogen peroxide in the cytosol. This process leads to ROS production and subsequently causes oxidative stress after depletion of GSH. The differences between Pt particles may be related to the diverse number of Pt atoms on the particles’ surface or various surface-to-volume NP ratios. Conversely, the alterations between particles can be an advantage during any future development of Pt-based anticancer NPs. As any dissolution and intranuclear location was observed for Au@Pt, application of PtNPs in Auger electron therapy seems to be more promising due to its successful penetration of the nuclear envelope and possibility of direct radiation-induced damage of DNA. Certainly, a particular application of PtNPs and Au@Pt particles might be placed in personalized cancer therapy with the reference to the tumor size and location, oxidation and angiogenesis.

## Figures and Tables

**Table 1:** Theoretical calculations for prediction of the differences in Au@Pt and PtNPs reactivity.

	Diameter *	Pt Atoms per NP **	SA:V	Pt Atoms on the Surface **	% of the Pt Surface Atoms
**Au@Pt**	30 nm (core) 9.70 nm (shell)	788,882	0.16	70,432	8.9%
**PtNPs**	2 nm	273	3.0	161	61%

* Platinum atomic radius considered to be 139 pm; ** theoretical calculations assuming spherical shape of nanoparticles. The shape was confirmed with HR-TEM (Au@Pt) and TEM (PtNPs) imaging. Data reported in Supplementary Information (Figure S1A,C).

## Data Availability

Not applicable.

## Supplementary Materials

The following supporting information can be downloaded at: https://www.mdpi.com/article/10.3390/ijms232314773/s1. References [31,38,39,40,41] are cited in Supplementary Materials.
