# Supplementary material for: Oxidative Status as an Attribute for Selective Antitumor Activity of Platinum-Containing Nanoparticles against Hepatocellular Carcinoma
Source: Int J Mol Sci. 2022 Nov 25;23(23):14773. doi: 10.3390/ijms232314773 (PMC9736793; doi:10.3390/ijms232314773)
Supplement: Supplementary file 1 [file ijms-23-14773-s001.zip › ijms-2028832-supplementary.pdf]

Oxidative Status as an Attribute for Selective Antitumor Activity of Platinum-Containing Nanoparticles against Hepatocellular Carcinoma

Kamil Wawrowicz [1], Agnieszka Majkowska-Pilip [1,2], Marzena Szwed [3], Kinga Żelechowska-Matysiak [1], Ewelina Chajduk [4], and Aleksander Bilewicz [1,*]

1. Centre of Radiochemistry and Nuclear Chemistry, Institute of Nuclear Chemistry and Technology, Dorodna 16 St., 03-195 Warsaw, Poland
2. Department of Nuclear Medicine, Central Clinical Hospital of the Ministry of the Interior and Administration, Wołoska 137 St., 02-507 Warsaw, Poland
3. Department of Medical Biophysics, Faculty of Biology and Environmental Protection, University of Lodz, Pomorska 141/143 St., 90-236 Lodz, Poland
4. Laboratory of Nuclear Analytical Techniques, Institute of Nuclear Chemistry and Technology, Dorodna 16 St., 03-195 Warsaw, Poland

* Correspondence: a.bilewicz@ichtj.waw.pl

SUPPLEMENTARY INFO

RESULTS AND DISCUSSION

Characterization of investigated platinum particles. The schematic overview, TEM or HR-TEM images and physicochemical properties of investigated particles are shown in Figure S1. PtNPs were imaged with TEM and, based on these measurements, the size distribution of particles was calculated. Synthesized NPs are spherical, homogenous, and highly dispersed (Figure S1/A), with a mean diameter based on the Gaussian distribution assessed at 1.94 ± 0.38 nm (Figure S1/B).Core-shell nanoparticles were characterized with HR-TEM to visualize distribution of the Pt shell of the AuNP core. Images confirmed the uniform arrangement of Pt atoms and the mean diameter was calculated as 39.02 ± 0.75 nm (Figure S1/C and D) [1].

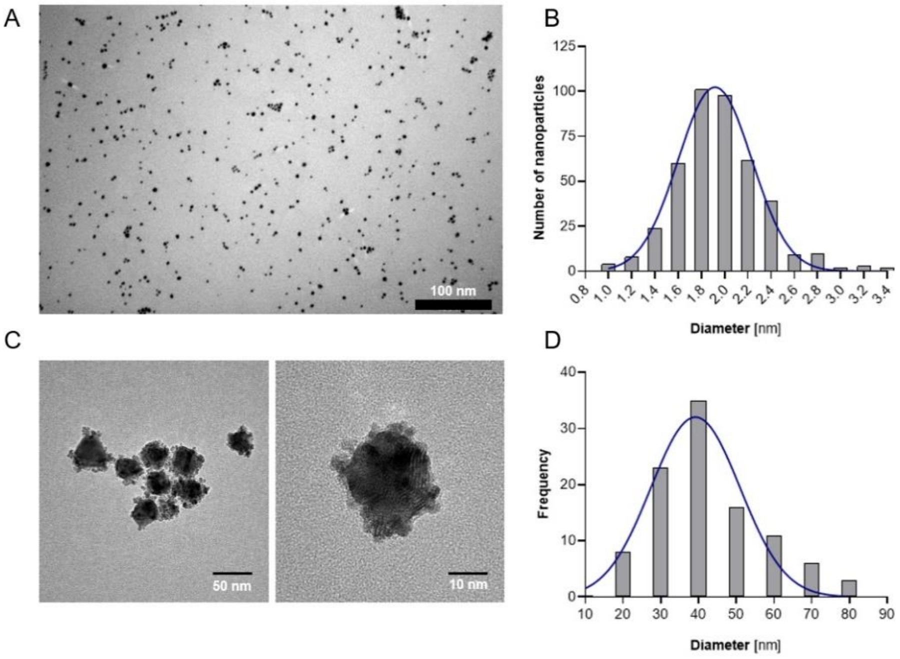

Figure S1. Physicochemical properties of PtNPs and Au@Pt: TEM image of 2 nm PtNPs. Spherical shape and high dispersity was confirmed during imaging (**A**); Gaussian distribution of the size of PtNPs – mean diameter was calculated as 1.94 ± 0.38 nm (n = 422) (**B**); HR-TEM images of platinum shell distribution on AuNP core – uniform arrangement of Pt shell was observed (**C**); Gaussian distribution of the size of Au@Pt calculated from dynamic light scattering (DLS) – mean diameter was calculated as 39.02 ± 0.75 (n = 5, for five independently synthesized nanoparticles solutions) (**D**).

HER2-targeted bioconjugates of Au@Pt and PtNPs and their cellular uptake. Herein, we report results concerning the linkage of PEG-trastuzumab to PtNPs with reference to the study performed with Au@Pt-PEG-[^{131}I]trastuzumab bioconjugate. A successful conjugation of trastuzumab to PtNPs, was achieved *via* a peptide bond between the free NH_2 group of lysine on the antibody heavy chain (Fc fragment) and N-hydroxysuccinimide (NHS) ester that is reactive toward primary amines. Bioconjugation, with using high affinity of thiol groups (in PEG) to Noble metals, proceeded with high (over 85%) yield and developed a nanosystem that contained per one PtNP around two molecules of trastuzumab (calculated with radiometric assays with - [^{131}I]trastuzumab), similarly as reported previously for Au@Pt. [1].

In order to determine whether Au@Pt- and PtNPs- trastuzumab conjugates bind specifically to HER2 receptors and are transported into the cell, we performed receptor binding affinity and internalization assessments. We used a radio a radiometric receptor binding assay and found Pt nanoparticles were conjugated with ^{131}I-trastuzumab. This study employed two different HER2-positive cell lines, namely BT474 breast cancer cells and SKOV-3 ovarian cancer cells, although the former have more HER2 transmembrane receptors than the latter (~1.9x10^6 and ~1.4x 10^6 respectively) [2]. Experiments were also carried out on the MDA-MB-231 cell line that lacks HER2 receptors [3],[4].

Synthesized bioconjugates bind specifically to HER2 receptors. Our observation was confirmed by a decrease of binding during receptor blocking with excess trastuzumab This phenomenon was not present in MDA-MB-231 cells that were used as a negative control.

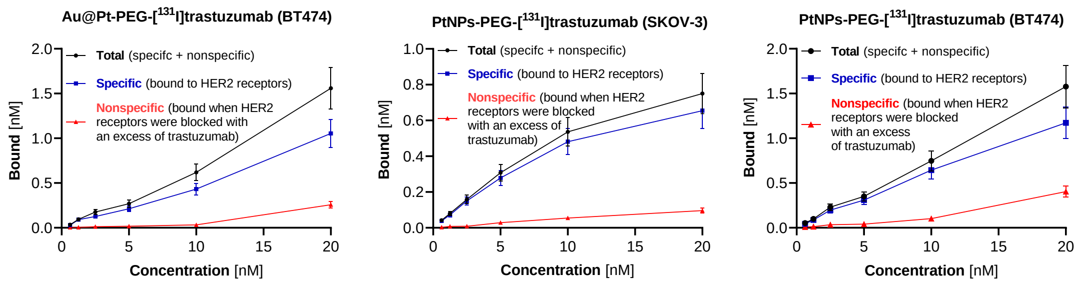

Figure S2. Receptor binding affinity curves of synthesized bioconjugates with SKOV-3 and BT474 cells. Evaluation of Au@Pt-PEG-[^{131}I]trastuzumab in SKOV-3 cells was previously reported in our manuscript. Decrease of binding during blocking the receptors with excess of trastuzumab (red line) proves specific binding of Au@Pt/PtNPs bioconjugates to HER2 receptors.

To gain further insight into intracellular uptake, we tested whether receptor binding of NPs is directly related to their internalization. Indeed, both SKOV-3 and BT474 cancer cell lines demonstrated a significant ($p < 0.0001$) internalization of bioconjugates, with over 80% of bioconjugates internalized during the first hour of treatment. The high uptake of Pt-and Au@Pt-based bioconjugates was observed until 24 h of incubation. Interestingly, if we compare the internalization ratio of PtNP bioconjugates into BT474 and SKOV-3 cancer cells, some variation in the data is present. The bioconjugate internalization ratio in SKOV-3 cells was higher than the BT474 cell line. These differences may be related to the altered physiology and variable density of HER2 receptors on the cellular surface.

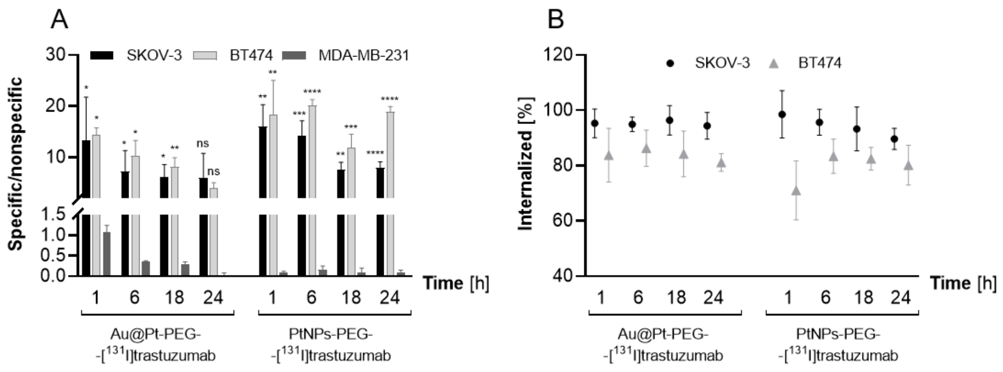

Figure S3. Receptor binding affinity studies of Au@Pt-PEG-[^{131}I]trastuzumab and PtNPs-PEG-[^{131}I]trastuzumab (**A**); Specific to nonspecific binding ratio shows significant ($p < 0.05$) between HER2+ cell lines (black and light grey bars) and HER2- cells (dark grey bar). Percentage of internalized bioconjugates in different time periods is presented in part (**B**). Statistical analysis were performed with using MDA-MB-231 cell line as control.

Cytotoxicity evaluation after incubation of cancer cells for up to 24 and 48h respectively with the different Pt particles and their bioconjugates.

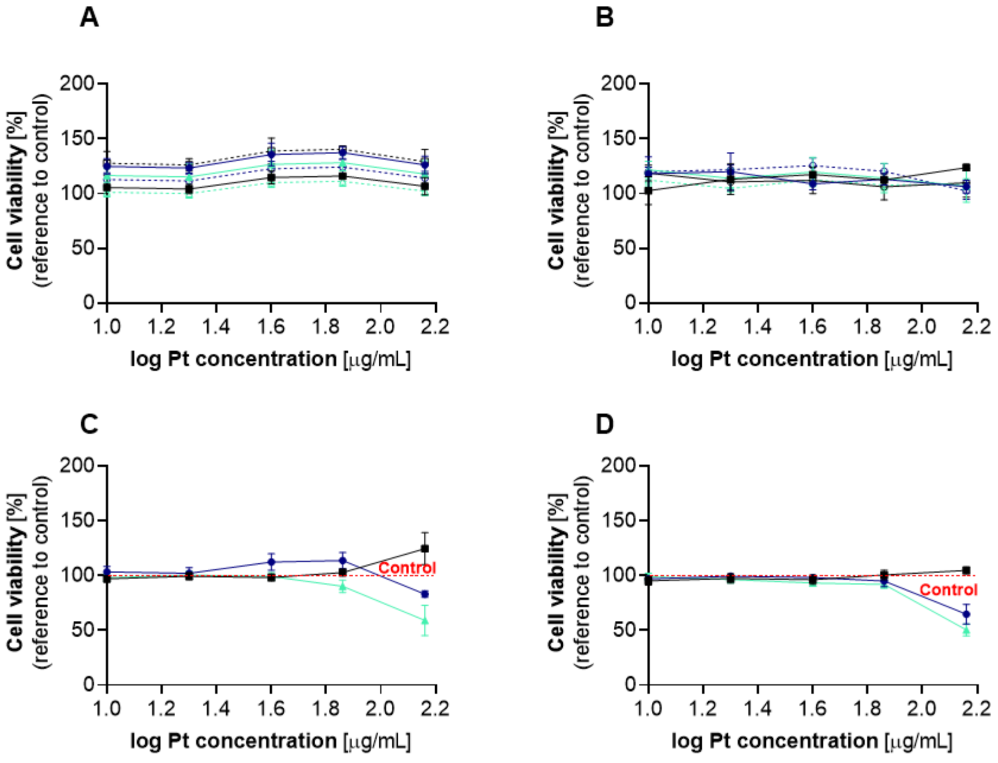

Figure S4. Cell viability curves of SKOV-3 (A.B) and HepG2 (C,D) treated with nanoparticles and its bioconjugates; A – SKOV-3/24h; B – SKOV-3/48h; C – HepG2/24h; D – HepG2/48h.

Fluorescence intensity from the oxidation of the $DFCDAH_2$ probe at the beginning of experiment was three times higher in HepG2 cells in comparison to SKOV-3 cells ($p < 0.0001$). In contrast, a 1.6-fold higher level of GSH was observed in SKOV-3 cells (28 nM/µg of protein) in comparison to HepG2 cells (17.50 nM/µg of protein). Simultaneously, we also measured ROS generation, in both cancer cell lines by inducing oxidative stress with 100 µM of H_2O_2. The results obtained demonstrated a negligible increase in ROS level, while in hepatic cells signal was more than twice higher in HCC cells. This corresponds with GSH and $DFCDAH_2$ oxidation levels in untreated cells, what indicates higher antioxidative resistance of HER2+ cells when compared to HCC.

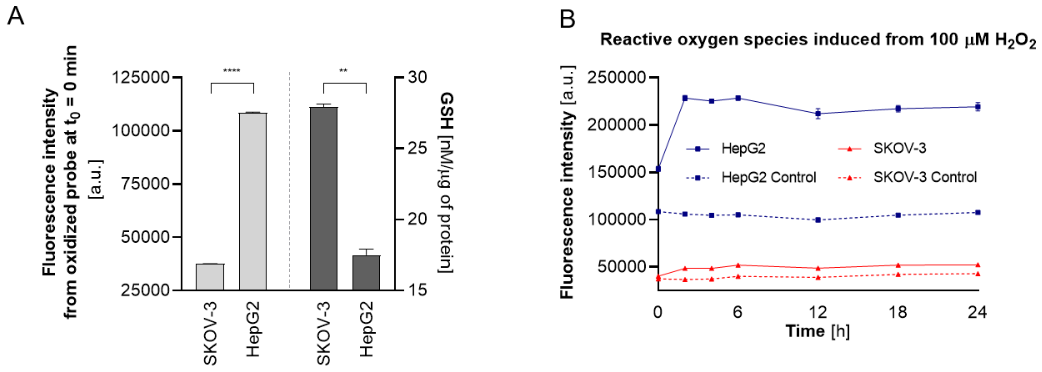

Figure S5. Fluorescence intensity of $DFCDAH_2$ probe and GSH level in untreated SKOV-3 and HepG2 cells (**A**). SKOV-3 cells showed lower probe oxidation and higher GSH level in reference to HepG2 cells. Reactive oxygen species induced from 100 µM H_2O_2 in SKOV-3 and HepG2 cells (**B**). Only HepG2 cells were susceptible for prooxidative action of hydrogen peroxide. This indicates, that SKOV-3 cells are more resistant to oxidative stress than HepG2.

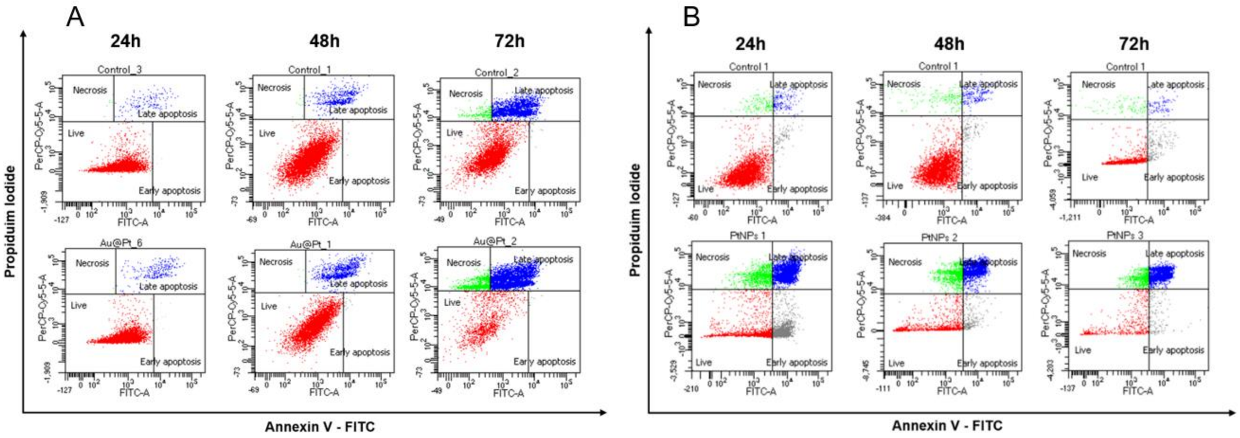

Figure S6. Annexin V assay in HepG2 cells after platinum nanoparticles treatment – dot plots of flow cytometry apoptosis analysis of cells treated with Au@Pt (**A**) and PtNPs (**B**) – red (**live**); grey (**early apoptotic**); blue (**late apoptotic**); green (**necrotic**). Upper dot blots (A, B) describe untreated control cells. Pt concentration used for this assay was 145 µg/mL.

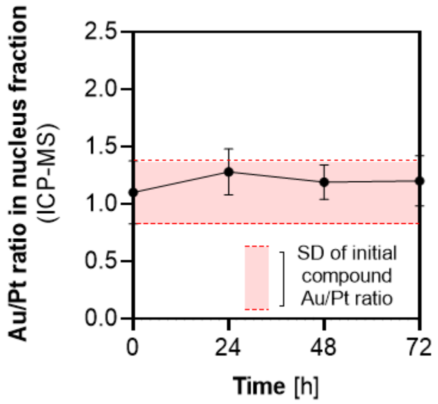

Figure S7. Au:Pt ratio in nuclei isolated fraction after incubation of HepG2 cells with Au@Pt. There were no significant changes in the Au:Pt ratio after each time incubation and the ratio was in the range of the initial (prior after synthesis) compound, what is marked with red area in graph. Thus, we concluded that any increased nuclear uptake of Au@Pt did not occurred, due to any changes in Au:Pt ratio, which should be decreased when compared to initial compound.

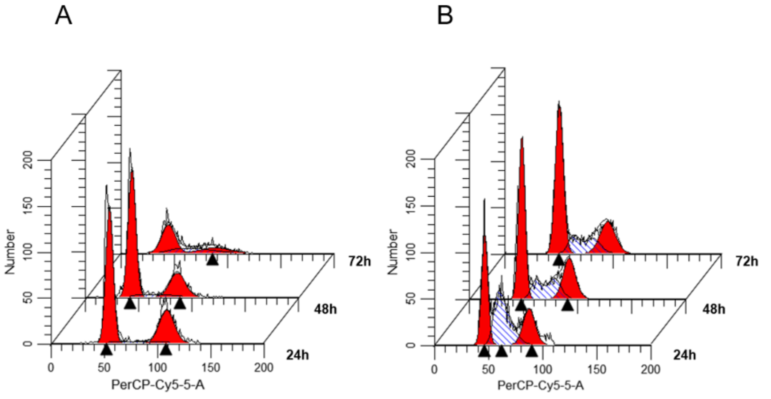

Figure S8. The blockage of HepG2 cell cycle induced with Au@Pt and PtNPs: Histograms of cell cycle arrest analysis after treatment with Au@Pt (**A**) and PtNPs (**B**); Pt concentration used for this assay was 145 µg/mL.

MATERIALS AND METHODS

Chemical reagents used. The chemical reagents were used as follows: hexachloroplatinic acid hexahydrate ($H_2PtCl_6{\cdot}6H_2O$); platinum chloride ($PtCl_2$); sodium rhodizonate dibasic 97% ($C_6Na_2O_6$); sodium carbonate (Na_2CO_3); sodium hydrogen carbonate ($NaHCO_3$); sodium chloride (NaCl); sodium hydroxide (NaOH); potassium hydroxide (KOH); hydrochloric acid (HCl); ascorbic acid ($C_6H_8O_6$); thiolated carboxylic-polyethylene 4α;-sulfanyl-ω carboxy PEG (HS-PEG-COOH, 5 kDa); N-acetyl-L-cysteine (NAC);, 3-Hydroxy-1,2-dimethyl-4(1H)-pyridone ($C_7H_9NO_2$); N-[(3-chlorophenyl)methyl]spiro[4H-quinoxaline-3,4′-piperidine]-2-amine ($C_{19}H_{21}ClN_4$);, 3-Amino-4-cyclohexylaminobenzoic acid ethyl ester ($C_{15}H_{22}N_2O_2$); hydrogen peroxide 30% (H_2O_2); L-Glutathione reduced; L-buthionine-sulfoximine (L-BSO); bovine serum albumin (BSA); L-ascorbic acid; N-ethylmaleimide (NEM); ethylenediaminetetraacetic acid (EDTA); triton X-100; phosphoric acid (H_3PO_4); N-[(3-Chlorophenyl)methyl]-spiro[piperidine-4,2′(1′H)-quinoxalin]-3′-amine; sodium dithionite ($Na_2O_4S_2$); protease and phosphatase inhibitors; phthaldialdehyde reagent (OPA); methyl and ethyl alcohol (MeOH/EtOH); trichloroacetic acid (TCA) were purchased from Merck & Co., Inc. (Kenilworth, NJ, USA). Diethylenetriaminepentaacetic acid (DTPA) was purchased from ChemaTech (Dijon, France). Orthopyridyldisulfide-polyethyleneglycol-succinimidyl carboxymethyl ester (OPSS-PEG-NHS, 5 kDa) was purchased from Creative PEGworks (Chapel Hill, NC, USA). Chloromethyl derivative of H_2DCFDA (CM-H_2DCFDA); annexin-V FITC conjugate; annexin-V binding buffer; RNase A, DNase and protease-free; iodogen (1,3,4,6-tetrachloro-3R,6R-diphenylglycouril); Pierce™ BCA Protein Assay Kit; hoechst 33258 and propidium iodide were purchased from Thermo Fischer Scientific (Waltham, MA, USA). Trastuzumab was isolated form Herceptin® (Roche Pharmaceuticals, Basel, Switzerland), Ogivri® (Mylan Pharmaceuticals, Morgantown, WV, USA) and Kanjinti® (Amgen, Center Drive Thousand Oaks, CA, USA).

For biological experiments following materials were used: growing medias - McCoy’s 5A, DMEM, EMEM; RPMI 1640; Trypsin EDTA solution C; water, cell culture grade; phosphate-buffered saline (PBS) and fetal calf serum from Biological Industries (Beth Haemek, Israel). McCoy’s 5A and EMEM *w/o* phenol red were purchased from Cytiva (Marlborough, MA, USA). Dimethylsulfoxide (DMSO) and the CellTiter96® AQueous One Solution Reagent (MTS compound) from Promega (Mannheim, Germany). SKOV-3, MDA-MB-231, BT-474 and HepG2 cells were obtained from the American Type Tissue Culture Collection (ATCC, Rockville, MD, USA) and cultured accordingly to the ATCC protocol. For experimental applications, over 80% confluent cells were used. All solutions were prepared with using ultrapure deionized water (18.2 MΩ·cm, Hydrolab, Straszyn, Poland). Stability control of [^{131}I]trastuzumab during the iodination was achieved by thin layer chromatography (TLC) technique with the use of Storage Phosphor System Cyclone Plus (Perkin Elmer, Waltham, MA, USA), glass microfiber chromatography paper impregnated with silica gel (iTLC SG, Agilent Technologies, Santa Clara, CA, USA) and with PBS buffer as mobile phase. Purification of iodinated antibody was performed with PD-10 Spehadex G25 desalting column purchased from Cytiva (Marlborough, MA, USA).

Radionuclides. For radioiodination of trastuzumab, ^{131}I (β^-, $t_{1/2}$ = 8.01 d, γ = 364 keV (81.50%) radionuclide was applied. Na^{131}I (n.c.a. - no-carrier-added, with the specific activity of 550 GBq/mg) was obtained from POLATOM Radioisotope Centre (Świerk, Poland).

For synthesis of Au@[^{197}Pt]Pt and [^{197}Pt]PtNPs 100 mg of ^{nat}Pt ($PtCl_2$) target was activated for with 1-2x10^4 n·cm^{2}·s^{-1} neutron flux for 6h followed by 4h cooling. Target material was dissolved with 5 mL of cc. HCl and 1 mL of hydrogen peroxide for 3h. After complete dissolution, reaction mixture was evaporated almost to dryness and ^{197}Pd (β^-, $t_{1/2}$ = 18 h, γ = 77 keV (17%); 191 keV (3,7%)) was suspended in 500 µL of 0.1M HCl. Further synthetic steps were conducted with the same procedure as described for non-radioactive nanoparticles.

Characterization techniques of nanoparticles. The size and shape of ultra-small, 2 nm PtNPs was confirmed with transmission electron microscopy (TEM) using the JEM 1400 (JEOL Co., Japan, 2008). Core-shell Au@Pt nanoparticles were visualized by high resolution TEM (HR-TEM) microcopy (TALOS™ F200X, Thermo Fischer Scientific-Waltham, MA, USA).

Synthesis and characterization of nanoparticles. PtNPs were synthesized in aqueous solution from acidic ($H_2PtCl_6{\cdot}6H_2O$) or salt precursor ($PtCl_2$) or [^{197}Pt]$PtCl_2$ with sodium rhodizonate as the reducing and stabilizing agent. The procedure was similar as reported elsewhere [5] with slight modifications. Briefly, 19.50 mg of Pt was dissolved in 20 mL of ultrapure water and heated for 30 min at 100°C with vigorous stirring. Then, 83.50 mg of sodium rhodizonate (48.5 mM in H_2O) was injected into the mixture and the solution rapidly changed color from light yellow to brownish. To ensure complete reduction of Pt^{4+} ions, the mixture was kept under stirring and heating for next 20 minutes, then slowly cooled to room temperature. For TEM imaging, 1 mL of PtNPs was filtrated *via* syringe and filter (0.22 µm) (Avantor, Radnor, PA, USA). PtNP were stored at 4°C in the dark with no loss of function for at least six months.

Core-shell nanoparticles used in this research were synthesized and characterized according to our previous protocol [1]. For all experiments presented herein, any modifications were implemented.

Synthesis of PtNPs-PEG-COOH and PtNPs-PEG-trastuzumab bioconjugate. Conjugates (PtNPs-PEG-COOH) and bioconjugates (PtNPs-PEG-trastuzumab) were synthesized according to a previously published protocol [1]. In brief, PEGylated PtNPs were prepared by adding 100-molar excess of PEG and mixed for 30 min. For bioconjugate synthesis, trastuzumab (200 µg) reacted with 25-molar excess of OPSS-PEG-NHS (5 kDa) in 100 mM sodium carbonate-bicarbonate buffer (pH ~ 8.90) overnight at RT. Unconjugated OPSS-PEG-NHS was purified with the use of centrifugal concentrators Vivaspin®500 to a 100 kDa cut-off (Sartorius, Goettingen, Germany). Subsequently, 100 µL of PtNPs were mixed with 80 µL OPSS-PEG-trastuzumab and 200 µL 20 mM sodium carbonate-bicarbonate buffer (2 mg/mL) and a 30 min conjugation was performed. Next, HS-PEG-COOH (100 mg/mL) were added for improved stability and for 45 minutes. Purification was performed with Vivaspin®500 100 kDa cut-off (10 min, 8000 rpm), and any unconjugated PtNPs were found to be membrane-permeable due to its MW (~50 kDa). Trastuzumab-conjugated PtNPs were dispersed in 400 µL of deionized water.

Internalization studies of PtNPs-PEG-trastuzumab bioconjugate. For internalization studies, all three cancer cell were seeded into 6-well plates (6-8 x 10^5 per well and incubated overnight. After incubation media was replaced with 5 nM of PtNPs-PEG-[^{131}I] trastuzumab dissolved in standard media and incubated for 1 h at 4°C to avoid internalization. Media was removed and cells were washed in PBS. Both media and PBS were stored as the unbound fraction and 1 mL of supplemented medium was added to the wells. Internalization ratio was measured at 1 h, 6 h, 18 h and 24 h. Two separated fractions were collected, namely membrane bound, but non internalized (1) and internalized (2). To collect the non-internalized fraction, 50 mM glycine·HCl (pH ~2.80) on ice was used as the receptor-bound antibody is not acid resistant and can be easily removed from cell membranes. All measurements and calculations were performed similarly as described above. Results presented (mean with SD) contains data from two individual experiments, with three repeats of each sample.

[1] Wawrowicz, K.; Majkowska-Pilip, A.; Gaweł, D.; Chajduk, E.; Pieńkowski, T.; Bilewicz, A. Au@Pt Core-Shell Nanoparticle Bioconjugates for the Therapy of HER2+ Breast Cancer and Hepatocellular Carcinoma. Model Studies on the Applicability of ^{193m}Pt and ^{195m}Pt Radionuclides in Auger Electron Therapy. *Molecules* **2021**, *26*, 2051. https://doi.org/10.3390/molecules26072051.

[2] Onsum, M.D.; Geretti, E.; Paragas, V.; Kudla, A.J.; Moulis, S.P.; Luus, L.; Wickham, T.J.; McDonagh, C.F.; MacBeath, G.; Hendriks, B.S. Single-cell quantitative HER2 measurement identifies heterogeneity and distinct subgroups within traditionally defined HER2-positive patients. *Am. J. Pathol.* **2013**, *183*, 1446–1460. https://doi.org/10.1016/j.ajpath.2013.07.015.

[3] Dai, X.; Cheng, H.; Bai, Z.; Li, J. Breast cancer cell line classification and Its relevance with breast tumor subtyping. *J. Cancer* **2017**, *8*, 3131–3141. https://doi.org/10.7150/jca.18457.

[4] Hua, W.; Christianson, T.; Rougeot, C.; Rochefort, H.; Clinton, G.M. SKOV3 ovarian carcinoma cells have functional estrogen receptor but are growth-resistant to estrogen and antiestrogens. *J. Steroid Biochem. Mol. Biol.* **1995**, *55*, 279–289. https://doi.org/10.1016/0960-0760(95)00187-5.

[5] Islam, M.T.; Saenz-Arana, R.; Wang, H.; Bernal, R.; Noveron, J.C. Green synthesis of gold, silver, platinum, and palladium nanoparticles reduced and stabilized by sodium rhodizonate and their catalytic reduction of 4-nitrophenol and methyl orange. *New J. Chem.* **2018**, *42*, 6472–6478. https://doi.org/10.1039/c8nj01223g.
